# FTY720, a sphingosine analog, altered placentome histoarchitecture in ewes

**DOI:** 10.1186/s40104-019-0411-0

**Published:** 2020-01-06

**Authors:** Kathrin A. Dunlap, Bryan G. White, David W. Erikson, M. Carey Satterfield, Christiane Pfarrer, Guoyao Wu, Fuller W. Bazer, Robert C. Burghardt, Kayla J. Bayless, Greg A. Johnson

**Affiliations:** 1 Department of Animal Science, Texas A&M University, College Station, TX 77843 USA; 2 Okanagan College Salmon Arm Campus, Salmon Arm, British Columbia Canada; 3 Endocrine Technologies Core, Oregon National Primate Research Center, Oregon Health & Science University, Beaverton, OR USA; 4 Department of Anatomy, University of Veterinary Medicine Hannover, Bischofsholer Damm 15, 30173 Hannover, Germany; 5 Department of Veterinary Integrative Biosciences, College of Veterinary Medicine and Biomedical Sciences, Texas A&M University, College Station, TX 77843 USA; 6 Department of Molecular and Cellular Medicine, Texas A&M Health Science Center, College Station, TX 77843 USA

**Keywords:** Placentome, Pregnancy, Sheep, Sphingosine1 phosphate (S1P)

## Abstract

**Background:**

The lysosphingolipid, sphingosine-1-phosphate, is a well-described and potent pro-angiogenic factor. Receptors, as well as the sphingosine phosphorylating enzyme sphingosine kinase 1, are expressed in the placentomes of sheep and the decidua of rodents; however, a function for this signaling pathway during pregnancy has not been established. The objective of this study was to investigate whether sphingosine-1-phosphate promoted angiogenesis within the placentomes of pregnant ewes. Ewes were given daily jugular injections of FTY720 (2-amino-2[2-(− 4-octylphenyl)ethyl]propate-1,3-diol hydrochloride), an S1P analog.

**Results:**

FTY720 infusion from days 30 to 60 of pregnancy did not alter maternal organ weights nor total number or mass of placentomes, but did alter placentome histoarchitecture. Interdigitation of caruncular crypts and cotyledonary villi was decreased, as was the relative area of cotyledonary tissue within placentomes. Also, the percentage of area occupied by cotyledonary villi per unit of placentome was increased, while the thickness of the caruncular capsule was decreased in ewes treated with FTY720. Further, FTY720 infusion decreased the number and density of blood vessels within caruncular tissue near the placentome capsule where the crypts emerge from the capsule. Finally, FTY720 infusion decreased asparagine and glutamine in amniotic fluid and methionine in allantoic fluid, and decreased the crown rump length of day 60 fetuses.

**Conclusions:**

While members of the sphingosine-1-phosphate signaling pathway have been characterized within the uteri and placentae of sheep and mice, the present study uses FTY720 to address the influence of S1P signaling on placental development. We present evidence that modulation of the S1P signaling pathway results in the alteration of caruncular vasculature, placentome architecture, abundance of amino acids in allantoic and amniotic fluids, and fetal growth during pregnancy in sheep. The marked morphological changes in placentome histoarchitecture, including alteration in the vasculature, may be relevant to fetal growth and survival. It is somewhat surprising that fetal length was reduced as early as day 60, because fetal growth in sheep is greatest after day 60. The subtle changes observed in the fetuses of ewes exposed to FTY720 may indicate an adaptive response of the fetuses to cope with altered placental morphology.

## Background

Successful placental development is necessary for embryonic survival and proper fetal growth, is dependent on vascularization of uterine and placental tissues, and has long-term implications for adult health [1–3]. The ruminant placenta is organized into discrete regions called placentomes. Approximately 90% of the blood from the uterine artery reaches these placentomes, which are critical for nutrient transfer from the maternal uterine circulation to the fetus (hemotrophic support) and exchange of gasses between these tissue compartments [4]. Placentomes develop highly branched placental chorioallantoic villi, termed cotyledons, that grow rapidly to interdigitate with maternal aglandular endometrial crypts, termed caruncles. Extensive angiogenesis extends blood vessels into the connective tissue of both cotyledonary villi and caruncular crypts to provide intimate juxtaposition of maternal and placental vasculatures [5]. Examination of the vascular architecture reveals a nearly 2-fold increase in endometrial capillary density by day 30 of gestation, and a 12-fold increase in capillary density within cotyledonary villi during the last two trimesters of pregnancy in sheep [6, 7]. Failure of placentome development results in fetal death [8].

Angiogenesis, the formation of new blood vessels from the existing vasculature [9], is mediated through several key signaling pathways, including prostanoid synthesis, angiotensin, integrins, metalloproteinases, and angiogenic growth factors. Vascular endothelial growth factor (VEGF) and basic fibroblast growth factor (bFGF) have well described roles in angiogenesis during gestation; however, another potent angiogenic factor, the lysosphingolipid sphingosine-1-phosphate (S1P), acts synergistically with VEGF and bFGF to increase angiogenic sprouting *in vitro* [10]. S1P is found in the bloodstream and is carried by high-density lipoproteins. S1P signaling proceeds through five high-affinity G-protein-coupled receptors termed S1P1 through S1P5 [11].

S1P receptors, as well as the sphingosine phosphorylating enzyme, sphingosine kinase 1 (SPHK1), are upregulated in the placentomes of sheep and the decidua of rodents [12, 13]. However, to date, a functional requirement for this signaling pathway during pregnancy has not been well established. The objective of this study was to investigate a possible role(s) for S1P in the promotion of angiogenesis within the placentomes of pregnant ewes. This was accomplished by analyzing the effects of jugular i.v. injection of FTY720 (2-amino-2[2-(− 4-octylphenyl)ethyl]propate-1,3-diol hydrochloride), which interacts with S1P1 receptors and acts as a pharmacological S1P antagonist [14], on ovine placentome development. We present evidence that modulation of the S1P signaling pathway results in the alteration of caruncular vasculature, placentome architecture, abundance of amino acids in allantoic and amniotic fluids, and fetal growth during pregnancy in sheep.

## Methods

### Donor ewes, embryo collection, and transfer

All experimental and surgical procedures were approved by the Institutional Agricultural Animal Care and Use Committee of Texas A&M University. Six multiparous Suffolk ewes were subjected to estrus synchronization using an Eazi-Breed Controlled Intravaginal Drug Releasing Device (CIDR) (Pharmacia & Upjohn Pty Limited, Rydalmere, New South Wales) for 12 days. Superovulation was achieved via twice daily (07:00 and 19:00 h) injections of follicle stimulating hormone) (Bioniche, Belleville, Ontario, Canada) over a four day period from days 9 to 13 after CIDR insertion with the dosage decreased daily (40, 30, 25 and 20 mg, respectively) for a total dosage of 115 mg. The CIDR was removed on the evening of day 12 and ewes were administered 20 mg Lutalyse (Pfizer, New York) i.m. and mated to four Suffolk rams over a 24 h period after detection of estrus.

Embryos (morulae/blastocysts) were collected from donor ewes by flushing the uteri on day 6 post-estrus. Briefly, feed and water were withheld from ewes for 24 h prior to anesthesia with 24 mg xylazine (Vedco, Inc. St. Joseph, MO). A 7 cm incision was made adjacent to the midline 5 cm below the mammary gland and the uterus was externalized. A Foley catheter (8 Fr, 5 cc balloon) was inserted into the uterine horn, and each horn was flushed independently with 30 mL of Vigro Complete Flush medium (AB Technology, Pullman, WA). Only high quality (Grade 1) morulae or blastocysts with an intact zona pellucida were used for the study.

Twenty multiparous Suffolk ewes were subjected to estrus synchronization via a 12-day treatment with a CIDR device. On day 12 the CIDR was removed and each ewe was administered 20 mg Lutalyse (Pfizer, New York) i.m. Estrus was detected by vasectomized rams fitted with a marking harness. One embryo per recipient ewe was transferred on day 6 post-estrus. Food and water were withheld from recipient ewes for 24 h prior to embryo transfer. Anesthesia was induced with 12 mg xylazine i.v. Upon exposure of the reproductive tract, the tip of the uterine horn ipsilateral to the ovary containing a CL was exteriorized so that a single grade 1 morula/blastocyst could be transferred into the uterine lumen of each recipient ewe. Pregnancy was assessed by ultrasonography on day 20 post-transfer (day 26 of pregnancy) using an Aloka SSD-900 V rectal probe with a 7.5 MHz linear array (Aloka, Wallingford, CT). All ewes were fed a diet meeting 100% of nutrient requirements as defined by current National Research Council Standards.

### Experimental design

Ewes confirmed pregnant via ultrasonography and were fitted with indwelling catheters inserted in the jugular vein. Ewes were assigned randomly to receive daily i.v. infusions from days 30 to 60 of pregnancy of either 5 mL saline vehicle (Control; *n* = 5) or 1.5 mg FTY720 in 5 mL saline (*n* = 6) (Husker Chemical, Shanghai, China). 1.5 mg/d was administered intravenously daily in each ewe, based on effective doses reported previously, where 1.25 and 5 mg/d doses in humans reduced central nervous system lesions and disease activity in multiple sclerosis patients [15]. FTY720 is derived from myriocin, a fungal metabolite of the Chinese herb *Iscaria sinclarii,* and is a structural analog of sphingosine that binds S1P receptors. FTY720 is also known as fingolimod [16].

### Tissue and fluid collection

Ewes were sacrificed on day 60 of pregnancy, and maternal organs removed. Specifically, maternal heart, liver, kidney, and spleen were removed, weighed prior to dissection, and samples from each were fixed in either fresh 4% paraformaldehyde in phosphate buffered saline (PBS, pH 7.2; Sigma, St. Louis, MO) or snap frozen in liquid nitrogen and stored at − 80 °C. The gravid uterus, dissected free of the ovary and mesometrium, containing uterus, placenta, fetus and fetal fluids was removed and weighed, and placentomes were counted and weighed [16, 17]. Several samples of placentomes from each uterine horn (thickness ~ 1–1.5 cm) were placed in fresh 4% paraformaldehyde fixative or snap frozen in Optimal Cutting Temperature Compound (Cat# 4583, Sakura Finetek, Torrance, CA). Tissues were fixed for 24 h in paraformaldehyde then transferred to 70% ethanol for 24 h, dehydrated through a graded ethanol series to xylene, and then embedded in Paraplast-Plus (Oxford Labware, St. Louis, MO).

Samples of allantoic and amniotic fluids were collected from each conceptus and aliquots stored at − 20 °C for subsequent analysis for amino acids. The fetus was excised from the placenta, weighed, measured for crown-rump length, and further dissected to obtain fetal heart, liver, kidneys, small intestine, and skeletal muscle. The weight of each organ was determined.

### Histological analyses

Paraffin-embedded placentomes were sectioned (5 μm), deparaffinized in CitraSolv (Fisher Scientific; Fairlawn, NJ) and rehydrated through a graded ethanol series to distilled water. Tissues were then exposed to either a Masson’s trichrome staining procedure as previously described in order to visualize collagen-containing connective tissue [18] or a Periodic acid-Schiff (PAS) staining procedure in order to visualize the basement membranes of blood vessels (Cat# 24200–1, Polysciences, Warrington, PA). Briefly, slides were incubated for 5 min in 0.5% periodic acid, washed three times with distilled water, and incubated in Schiff’s reagent for 15 min. Slides were then washed 2 times in 0.55% potassium metabisulfite to remove reagent, and placed under running water for 10 min. A counterstain of hematoxylin was applied to the slides for 30 s, followed by rinsing in running tap water. Both Masson’s trichrome stained and PAS stained slides were then dehydrated through ethanol to xylene, and coverslips were fixed with Permount (Fisher Scientific, Fair Lawn, NJ).

Histoarchitectural measurements including caruncular capsule thickness, crypt thickness, and relative tissue area occupied by cotyledonary villi were performed using ten, 940 μm × 740 μm frames from ten nonsequential 5 μm placentome sections from each ewe [19, 20], and evaluated using an Axioplan 2 microscope (Carl Zeiss, Thornwood, NY) interfaced with an Axiocam HR digital camera and Axiovision 4.3 software (Carl Zeiss). Caruncular crypt identification (primary, secondary, or tertiary) was determined using previously defined criteria [21, 22]. Briefly, primary caruncular crypts were categorized as major septa that originate from the caruncular stalk with a stromal core that runs perpendicular to the caruncular capsule. Secondary crypts were defined as possessing smaller but still clearly visible stromal cores which branched from a defined primary crypt, or, in cases in which origin was not obvious, crypts that traversed the placentome more nearly parallel to the supporting capsule. Tertiary crypts extended perpendicularly from secondary crypts as smaller bud-like projections composed of stromal and vascular components. Assessment of crypt identity was performed in replicate 5200 μm × 4070 μm frames of ten nonsequential 5 μm thick placentome tissue sections.

Further morphometric analyses of syncytial length and caruncular stromal area of placentomes was performed on PAS-stained sections that were captured using an Axioplan 2 microscope (Carl Zeiss, Thornwood, NY) interfaced with an Axiocam HR digital camera and Axiovision 4.3 software (Carl Zeiss). Images from the deep (capsule side), middle, and shallow (fetal side) regions of placentomes (at least 5 images/area per animal) were captured. The area per image of shallow and middle regions of placentomes was 2.516 million μm^2^, and the area per image of deep regions was 608 μm^2^. Images were analyzed with the ImageJ software package (National Institutes of Health, Frederick, MD). Syncytial length was determined by tracing the interface between the maternal and fetal placentomal compartments and recording the average pixel length per image. Maternal stromal area was calculated by measuring the pixel area contained within the caruncular placentomal compartment and subtracting any vascular area within the stroma.

Placentome vascularity was assessed using VE-cadherin immunostained slides described in the following section. Briefly, VE-cadherin immunostained placentome slides were captured using the Axioplan 2 microscope system. Images from comparable regions within the deep (capsule side), middle, and shallow (fetal side) regions of placentomes (at least 7 images/area/animal) and vessel number were determined through a manual count of observable vessels in both the maternal and fetal placentome compartments. To assess vascular size, PAS-stained sections from paraffin-embedded tissue were used to image individual vessels at a frame size of 150 μm^2^. Using the ImageJ software package (National Institutes of Health, Frederick, MD), the circumference of individual vessels were traced to determine and record pixel area within individual vessels. These data were then used to calculate the average vessel size in maternal and fetal placentome compartments within each placentomal region (deep, middle, and shallow).

### Immunofluorescence analyses

For assessment of vessel number, immunofluorescence analysis of OCT-fixed placentomal samples was performed as previously described with some modifications [23]. Briefly, placentome sections (5 μm) were fixed in cold, 2% paraformaldehyde for 10 min at 4 °C, followed by washing in 1% Triton / 1X PBS for 10 min at 4 °C. After isolating sections with a hydroscopic barrier, sections were washed with PBS/Tween [0.02 mol/L (3%)] and blocked in 10% normal goat serum for 1 h at room temperature. Sections were again washed with PBS/Tween before incubating in VE-cadherin primary antibody solution (5 μg/mL of rabbit anti-VE-cadherin, #ALX-210-232-C100, Enzo Life Sciences) overnight at 4 °C. Proteins were detected by incubation with an Alexa Fluor 488-conjugated anti-rabbit secondary antibody (4 μg/mL; Cat #A-11008, Life Technologies, Grand Island, NY) at room temperature for 1 h. Slides were overlaid with a coverslip and Prolong antifade mounting reagent containing the nuclear counterstain DAPI (Cat #P36935, Life Technologies, Grand Island, NY).

Vimentin and cytokeratin 8.13 proteins were localized in frozen placentomes from each animal by immunofluorescence staining as previously described [24]. Briefly, tissue sections (10 μm) were fixed in − 20 °C methanol, washed in PBS containing 0.3% vol/vol Tween 20 in PBS, blocked in 10% vol/vol normal goat serum for 1 h at room temperature, and incubated overnight at 4 °C with the following antibodies: mouse anti-vimentin (5 μg/ml, #V6630, Sigma) or mouse anti-cytokeratin (5 μg/mL, #C6909, Sigma) or negative control (5 μg/mL, mouse IgG, Invitrogen). Tissue-bound primary antibody was then detected with goat anti-mouse IgG Alexa Fluor 488 (4 μg/mL). Slides were overlaid with Prolong antifade with DAPI (Invitrogen) and a cover glass.

VE-cadherin was co-localized with alpha smooth muscle actin (αSMA) in frozen placentome cross-sections of each animal by immunofluorescence staining as previously described [24]. Briefly, 10 μm sections were cut, fixed for 10 min in 2% paraformaldehyde at 4 °C, washed with 1% Triton X-100 in PBS for 10 min at 4 °C, and blocked as described above. After dipping in rinse solution at room temperature, sections were incubated overnight at 4 °C with the initial primary antibody (5 μg/mL of mouse anti-αSMA, #A2547, Sigma, or 5 μg/mL of mouse IgG). Following washes, sections were incubated with the initial secondary antibody for 1 h at room temperature (4 μg/mL of goat anti-mouse IgG Alexa Fluor 594, Invitrogen), washed, and incubated overnight at 4 °C with the second primary antibody (5 μg/mL of rabbit anti-VE-cadherin, or 5 μg/mL of rabbit IgG, Sigma) Following washes, sections were incubated with secondary antibody (4 μg/mL goat anti-rabbit IgG Alexa Fluor 488, Invitrogen), washed, dipped in distilled-deionized water, and overlaid with Prolong antifade mounting reagent.

### Amino acid analyses

Amniotic and allantoic fluids (0.5 mL) were deproteinized with an equal volume of 1.5 mol/L HClO_4_, followed by addition of 0.25 mL 2 mol/L K_2_CO_3_. The extract was then analyzed for amino acids by fluorometric HPLC methods involving pre-column derivatization with *o*-phthaldialdehyde as described previously [25]. The integration of chromatographic peaks was performed using Millenium-32 Software (Waters, Milford, MA).

### Photomicrography

Digital photomicrographs of Masson’s trichrome and immunofluorescence staining were obtained using the Axioplan 2 microscope system. For immunofluorescence microscopy, digital camera settings were evaluated to confirm the absence of ‘spectral bleed through’ between DAPI, Alexa Fluor 488, and Alexa Fluor 594 filter sets. In these studies, once the distribution of individual antigens was established, the co-distribution of two antigens was investigated simultaneously in individual sections using compatible primary and Alexa Fluor 488- or Alexa Fluor 594-secondary antibody combinations with appropriate filter sets. Individual fluorophore images along with images of DAPI stained nuclei were recorded sequentially with AxioVision 4.3 software and evaluated in multiple fluorophore overlay images recorded in Zeiss Vision Image file format, which were subsequently converted to Tagged Image File format. Photographic plates were assembled using Adobe Photoshop CS4 (Adobe Systems Inc., San Jose, CA). All sections from each day and treatment were assessed as a group, and sections exhibiting the most representative immunostaining pattern for each day and treatment were selected for inclusion in photographic plates.

### Statistical analyses

Data from morphometric analyses were normalized to overall pixel area of captured image prior to statistical analyses. Statistical significance among data sets was assessed by a two-tailed *t*-test (FTY720 vs. control), One-way ANOVA analysis (gestational profile), using GraphPad Prism version 5.0 (GraphPad Software, San Diego, CA, www.graphpad.com). Differences with probability values of *P* < 0.05 were considered significant, while *P* < 0.10 indicated a trend towards a significant effect. Data are presented as mean values and their standard errors (SE).

## Results

### FTY720 infusion from days 30 to 60 of pregnancy did not alter maternal organ weights

Total ewe weight and weight of maternal heart, kidney, spleen, and liver were not different (*P* > 0.10) between treatment groups (Table 1). Gravid uterine wet weight, which includes the uterus, placenta, fetus and fetal fluids, tended to be greater for ewes treated with FTY720 as compared to control ewes (*P* < 0.10) (Table 1); however, neither total number nor mass of placentomes differed between treatments (*P* > 0.10) (Table 2).

**Table 1:** FTY720 infusion did not alter maternal organ weights

Maternal measures	Control	FTY720	*P* value
Body weight, kg	148.89 ± 8.39	165.48 ± 8.07	0.19
Heart, g	376.8 ± 31.69	427.33 ± 28.93	0.27
Kidney, g	162.8 ± 11.85	180.33 ± 10.81	0.30
Spleen, g	134 ± 14.41	153.66 ± 13.16	0.33
Liver, g	858.4 ± 67.32	924.33 ± 61.46	0.48
Uterus, g	1125.6 ± 77.55	1332 ± 70.79	0.08

**Table 2:** FTY720 infusion did not alter placentome number nor mass

Placentome measures	Control	FTY720	*P* value
Placentome mass, g	212.4 ± 50	248.66 ± 45.67	0.61
Placentome number	44.2 ± 8.02	54.66 ± 7.32	0.36
Placentome average mass, g/placentome	5.01 ± 0.96	4.45 ± 0.87	0.68

### FTY720 infusion from days 30 to 60 of pregnancy decreased fetal length

Fetal mass did not differ between treatment groups at day 60 (*P* > 0.10); however, the crown-rump lengths of fetuses from vehicle treated ewes were greater than for fetuses from ewes receiving FTY720 (*P* < 0.05; Table 3). Weights of fetal heart, kidney, spleen, or liver were not affected by treatment (*P* > 0.10; Table 3).

**Table 3:** FTY720 infusion decreased fetal length, but did not affect organ weights

Fetal measures	Control	FTY720	*P* value
Crown-rump length, mm	327.66 ± 3.81	313.18 ± 3.03	0.02
Body weight, g	58.32 ± 2.25	59.21 ± 2.05	0.77
Heart, g	0.54 ± 0.03	0.57 ± 0.03	0.67
Kidney, g	0.75 ± 0.07	0.74 ± 0.06	0.9
Spleen, g	0.02 ± 0.003	0.02 ± 0.003	0.91
Liver, g	4.46 ± 0.29	4.48 ± 0.29	0.95

### FTY720 infusion from days 30 to 60 of pregnancy altered placentome histoarchitecture

**Fig. 1:**
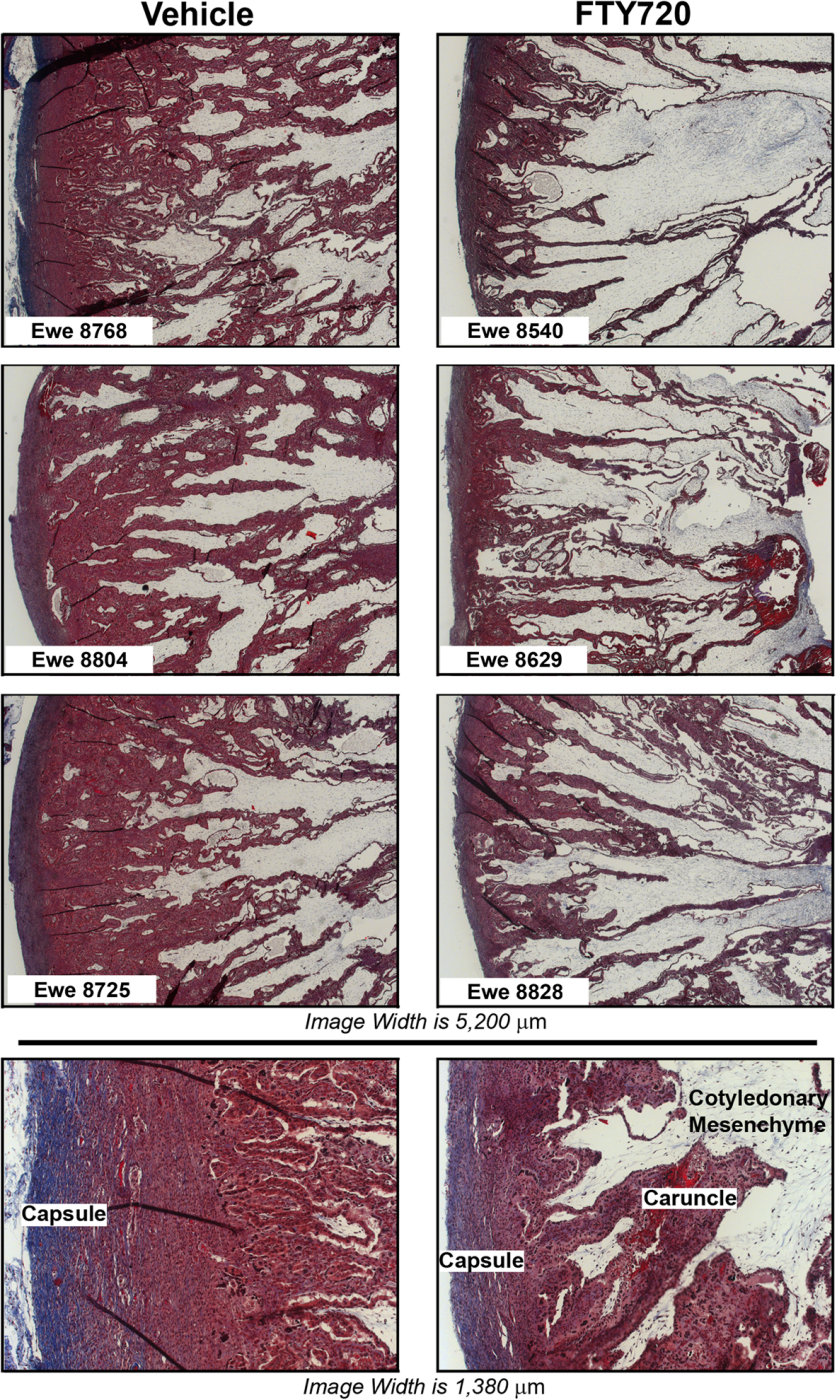
Assessment of the Histoarchitecture of placentomes using Masson’s Trichrome stain (nuclei, black; cytoplasm and muscle fibers, red; extracellular matrix components, blue). Upper six panels; three representative placentomes from control (Vehicle) and FTY720 treated ewes (width of each image is 5200 μm). Lower two panels; representative higher magnification of placentomes from control and FTY720 treated ewes (width of each image is 1380 μm)

**Fig. 2:**
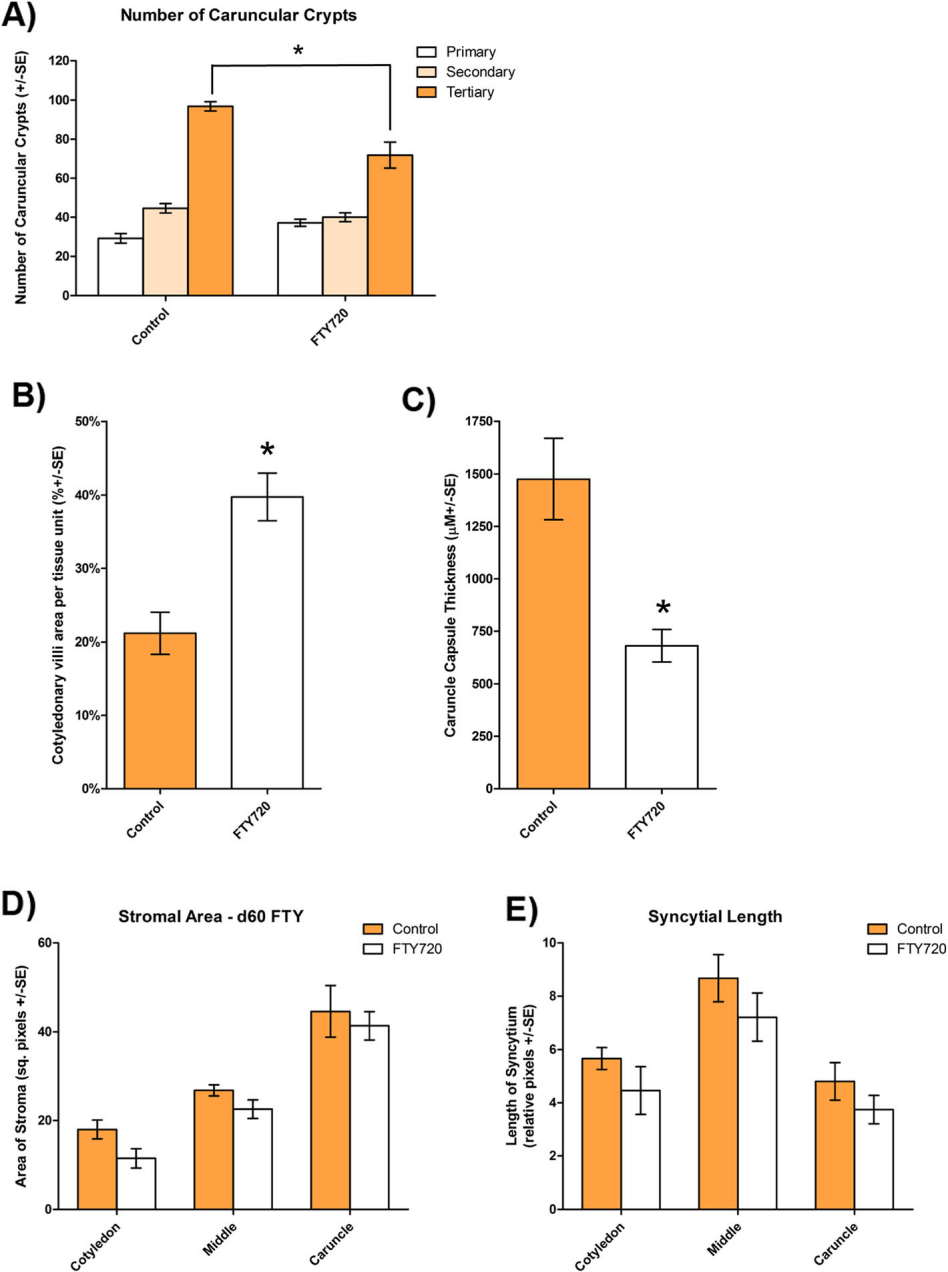
Morphometric analysis of placentomes from control and FTY720 treated ewes. **a**) Branching of caruncular crypts. The panel compares the mean ± SE for the number of primary (white bars), secondary (pale orange bars), and tertiary (dark orange bars) caruncular crypts per unit in samples of placentome tissue from control and FTY720 treated ewes (* *P* < 0.05) **b**) Cotyledonary area per unit of placentome tissue. The fig. Compares the mean ± SE cotyledonary area per unit of placentome tissue from control (orange bars) and FTY720 (white bars) treated ewes (* *P* < 0.05). **c**) Caruncular capsule thickness. The panel compares the mean ± SE for caruncular capsule thickness in placentomes from control (orange bars) and FTY720 (white bars) treated ewes (* *P* < 0.05). **d**) Caruncular stromal area per unit of placentome tissue. The panel compares the mean ± SE area of caruncular stromal tissue in samples of placentome tissue from control (orange bars) and FTY720 (white bars) treated ewes. Measurements were recorded from the deep (capsule side; Caruncle), middle, and shallow (fetal side; Cotyledon) areas of placentomes **e**) Length of syncytial epithelium. The panel compares the mean ± SE length of syncytial epithelium in samples of placentome tissue from control (orange bars) and FTY720 (white bars) treated ewes. Measurements were recorded from the deep (capsule side; Caruncle), middle, and shallow (fetal side; Cotyledon) areas of placentomes

**Fig. 3:**
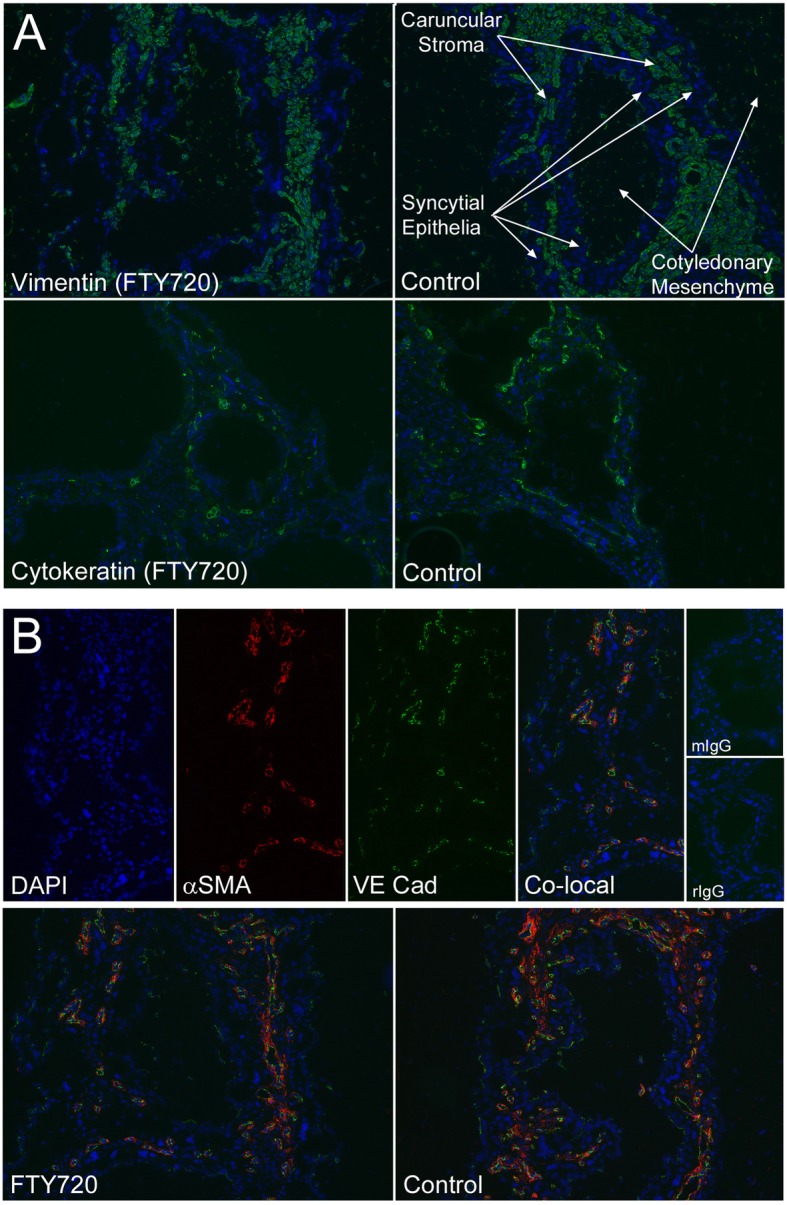
Caruncular and cotyledonary vasculature in placentomes from control and FTY720 treated ewes. **a**) Immunofluorescence staining (green) for vimentin and cytokeratin to detect connective tissue and syncytial epithelium within the caruncles and cotyledons of placentomes from control and FTY treated ewes. **b**) (Top Panels) Individual and co-localized (Co-local) fluorescence images detecting DAPI (blue), αSMA (red), and VE Cad (green) within the placentome of an FTY720 treated ewe; width of fields is 370 μm. Mouse immunoglobulin control, mIgG; rabbit immunoglobulin control, rIgG. (Bottom Panels) Co-localization of VE Cad (green) and αSMA (red) to blood vessels within the caruncles and cotyledons of control and FTY720 treated ewes; width of fields is 870 μm

Although FTY720 did not significantly affect placentome number or mass (Table 1; *P* > 0.10), it altered placentome morphology. FTY720 treatment decreased interdigitation of caruncular crypts and cotyledonary villi while increasing the relative area of cotyledonary tissue within placentomes as assessed after using Masson’s Trichrome stain (Fig. 1). The degree of caruncular crypt branching differed between control and FTY720 placentomes. FTY720 treated samples possessed fewer tertiary crypts than controls per unit of tissue (*P* < 0.05); however, there were no differences in the number of primary or secondary crypts between treatment groups (*P* > 0.1) (Fig. 2a). Concommittant with the decreased caruncular crypt branching within the placentomes in FTY720 treated ewes, there was a greater percentage area occupied by cotyledonary villi per unit of tissue when compared to placentomes in control ewes (*P* < 0.05) (Fig. 2b). Additionally, the shape of the tips of the maternal crypts (adjacent to the fetal chorionic side) differed between treatments. In control ewes, the tips were shaped like clubs or plates, while the tips of the crypts in FTY720 treated ewes remained thin (Fig. 1). Changes in placentome crypts and villi were accompanied by a decrease in the thickness of the caruncular capsule in ewes treated with FTY720 as compared to control ewes (*P* < 0.001) (Figs. 1 and 2c). FTY720 treatment of ewes did not alter (*P* > 0.10) the total area occupied by caruncular stroma within all anatomical regions of placentomes as compared to control ewes (Fig. 2d). Consistent with changes in the ratio of cotyledonary to caruncular tissue within placentomes and the decreased number of caruncular tertiary crypts, treatment of ewes with FTY720 had a tendency (*P* < 0.10) to decrease the length of the syncytial epithelium that forms the interface between caruncular and cotyledonary connective tissues (Fig. 2e). This tendency to decrease syncytial length was observed in all anatomical regions of placentomes. No clear differences in the microanatomy of the connective tissue or syncytial epithelium were observed in placentomes from control or FTY720 treated ewes based on immunofluorescence analyses and antisera directed to vimentin and cytokeratin (Fig. 3a).

### FTY720 infusion from days 30 to 60 of pregnancy reduced caruncular vasculature in placentomes

**Fig. 4:**
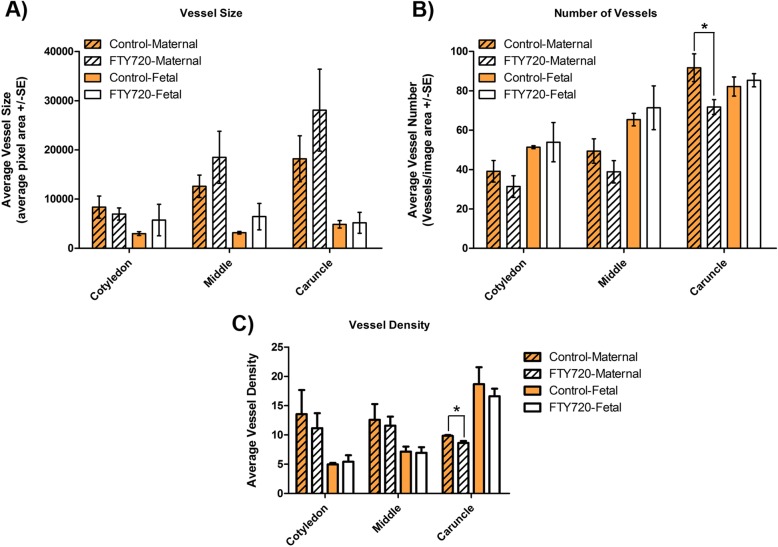
Size, number and density of blood vessels in placentomes from control and FTY720 treated ewes. **a**) Size of placentomal blood vessels. The panel compares the mean ± SE size of caruncular (Maternal) and cotyledonary (Fetal) blood vessels in samples of placentome tissue from control (orange bars) and FTY720 (white bars) treated ewes. **b**) Number of placentomal vessels. The panel compares the mean ± SE number of caruncular and cotyledonary blood vessels in samples of placentome tissue from control and FTY720 treated ewes (* *P* < 0.05). **c**) Density of placental vessels. The panel compares the mean ± SE average density of caruncular and cotyledonary blood vessels in samples of placentome tissue from control and FTY720 treated ewes. Legend: Cotyledon, shallow region of placentome near the fetal side; Caruncle, deep region of placentome near the capsule side; Middle, middle region of placentome (* *P* < 0.05)

Immunofluorescence co-localization of VE-cadherin and SMA in placentomes from control and FTY720 treated ewes demonstrated, as previously reported [26], that caruncular crypts contained arteries and arterioles with wide lumens and a significant tunica media layer conducive to maintaining a high rate and volume of blood flow, whereas cotyledonary villi contained primarily capillaries (Fig. 3b). To more closely examine whether FTY720 treatment altered the formation of blood vessels between treatment groups, morphometric analyses of PAS-stained placentome thin sections were performed. Although FTY720 treatment of ewes did not significantly affect the size of blood vessels in caruncular or cotyledonary regions of placentomes (Fig. 4a), FTY720 treatment decreased the total area occupied by blood vessels within caruncular villi (Fig. 4b and c) (*P* < 0.05). While FTY720 did not affect blood vessel size and density in any region of cotyledonary tissue, or in caruncular tissue near the fetal side and in the middle region of placentomes, FTY720 significantly decreased the number of blood vessels (Fig. 4b) as well as the density of blood vessels (Fig. 4c) within caruncular tissue near the placentome capsule i.e., near the base of the caruncular crypts where they emerge from the capsule.

### FTY720 infusion from days 30 to 60 of pregnancy altered amino acid profiles in fetal fluids

Evaluation of maternal plasma collected at gestational day 60 from control and FTY720 treated ewes revealed no significant differences in concentrations of all amino acids determined (data not shown). However FTY720 exposure altered the amino acid profiles in allantoic and amniotic fluids. In amniotic fluid, both aspartic acid (Asp) and glutamic acid (Glu) decreased in FTY720 treated ewes (*P* < 0.005; Table 4). In allantoic fluid, methionine (Met) was less abundant (*P* < 0.05) in FTY720 treated as compared to control ewes (Table 5). Recoverable levels of asparagine (Asn), serine (Ser), alanine (Ala), glutamine (Gln), beta-alanine (bAla), citrulline (Cit), arginine (Arg), lysine (Lys), histidine (His), glycine (Gly), threonine (Thr), taurine (Tau), tyrosine (Tyr), tryptophan (Trp), valine (Val), phenylalanine (Phe), isoleucine (Ile), leucine (Leu), ornithine (Orn), cysteine (Cys), and proline (Pro) in amniotic and allantoic fluids (Table 4 and Table 5) were not affected by treatment (*P* > 0.10).

**Table 4:** FTY720 infusion altered amino acid profiles in amtiotic fluid

Items	Amniotic fluid log-transformed data, nmol/mL		*P* value
	Control	FTY720	
ASP	1.20 ± 0.05	0.90 ± 0.05	0.002
GLU	3.90 ± 0.07	3.55 ± 0.06	0.004
ASN	3.83 ± 0.06	3.91 ± 0.05	0.335
SER	4.53 ± 0.11	4.69 ± 0.10	0.331
GLN	5.75 ± 0.05	5.70 ± 0.05	0.433
HIS	2.50 ± 0.10	2.68 ± 0.09	0.196
GLY	5.41 ± 0.13	5.56 ± 0.12	0.400
THR	4.71 ± 0.12	4.87 ± 0.11	0.327
CIT	4.51 ± 0.16	4.67 ± 0.14	0.496
ARG	5.35 ± 0.08	5.41 ± 0.08	0.557
b-ALA	3.42 ± 0.10	3.51 ± 0.09	0.515
TAU	4.54 ± 0.09	4.67 ± 0.08	0.290
ALA	6.61 ± 0.07	6.52 ± 0.06	0.344
TYR	3.95 ± 0.12	3.81 ± 0.11	0.420
TRP	2.52 ± 0.14	2.43 ± 0.13	0.698
MET	3.26 ± 0.11	3.06 ± 0.10	0.188
VAL	4.45 ± 0.09	4.33 ± 0.08	0.388
PHE	3.38 ± 0.11	3.19 ± 0.10	0.249
ILE	3.13 ± 0.07	3.07 ± 0.06	0.516
LEU	4.18 ± 0.08	4.14 ± 0.08	0.748
ORN	5.23 ± 0.10	5.21 ± 0.09	0.923
LYS	5.20 ± 0.11	5.37 ± 0.10	0.289

**Table 5:** FTY720 infusion altered amino acid profiles in allantoic fluid

Items	Allantoic fluid (log-transformed data), nmol/mL		*P* value
	Control	FTY720	
ASP	4.03 ± 0.36	3.97 ± 0.33	0.916
GLU	5.18 ± 0.50	5.06 ± 0.46	0.864
ASN	6.10 ± 0.32	5.79 ± .30	0.501
SER	7.41 ± 0.26	7.14 ± 0.24	0.460
GLN	8.67 ± 0.49	8.50 ± 0.44	0.810
HIS	5.49 ± 0.41	5.31 ± 0.37	0.742
GLY	7.92 ± 0.33	7.55 ± 0.30	0.422
THR	6.87 ± 0.33	6.44 ± 0.30	0.362
CIT	7.82 ± 0.31	7.54 ± 0.28	0.502
ARG	7.19 ± 0.22	6.92 ± 0.20	0.370
b-ALA	6.65 ± 0.32	6.23 ± 0.29	0.360
TAU	7.47 ± 0.33	7.24 ± 0.30	0.605
ALA	9.04 ± 0.43	8.68 ± 0.39	0.550
TYR	5.63 ± 0.22	5.18 ± 0.20	0.168
TRP	4.57 ± 0.20	4.10 ± 0.18	0.121
MET	4.60 ± 0.17	4.07 ± 0.15	0.042
VAL	5.96 ± 0.24	5.61 ± 0.22	0.291
PHE	4.95 ± 0.23	4.71 ± 0.21	0.456
ILE	4.75 ± 0.22	4.43 ± 0.20	0.317
LEU	5.89 ± 0.22	5.52 ± 0.20	0.240
ORN	7.58 ± 0.36	6.98 ± 0.33	0.249
LYS	7.15 ± 0.34	6.70 ± 0.31	0.351

## Discussion

While members of the S1P signaling pathway have been temporally and spatially characterized within the uteri and placentae of sheep and mice [13, 14], the present study uses FTY720 to address the influence of S1P signaling on placental develpment. We utilized pregnant sheep, a well-established model for placental/fetal development including the study of placental insufficiency [17, 20, 27, 28]. It is noteworthy that daily systemic administration of FTY720 from days 30 to 60 of pregnancy decreased the total area occupied by blood vessels within the caruncular villi (total number and density of vessels) of placentomes, increased the ratio of cotyledonary to caruncular tissue within placentomes and decreased the number of caruncular tertiary crypts within placentomes. Concomitant with these alterations in placentome morphology, fetal crown rump length was .modestly, but significantly, decreased on day 60, and Asp and Glu in amniotic fluid and Met in allantoic fluid were decreased. Although the physiological mechanism(s) through which fetal growth was altered remain to be determined, the findings of the present study provide insight into the physiological events that regulate placentome development in sheep, and suggest that alterations in the development of these structures, that are essential to nutrient transport to the fetus, may effect fetal growth.

The vascularization of endometrial caruncular crypts decreased with FTY720 treatment. It has been previously reported that total endometrial capillary surface area increases between days 18 and 30 in pregnant sheep, and dramatic increases in the placental vasculature follow in the second and third trimesters of pregnancy [6, 7]. Our data indicate a significant decrease in endometrial vasculaturization with no observed differences in placental vascularization at day 60. This decrease in endometrial angiogenesis correlated with altered placentome architecture, decreased Asp, Glu and Met in allantoic and/or amniotic fluids, and decreased fetal crown rump length. It is possible that the changes in placentome architecture observed in this study are a result of developmental compensation to to this decreased caruncular vascularization. The present *in vivo* study demonstrated that FTY720 treatment results in an increased ratio of cotyledonary to caruncular tissue within a placentome, concomitant with decreased caruncular crypt branching. In addition, caruncular crypts in FTY720 treated ewes were thin as compared to those for control ewes, and cotyledonary villi invaded more deeply into the crypts resulting in decreased thickness of the placentome capsule. It has been known for some time that the sheep placentome shows a high degree of plasticity in response to changes in the physiological environment [16], and that increased predominance of fetal tissue within the placentome occurs when ewes are subjected to nutrient restriction during the first half of pregnancy [17]. Steyn and colleagues [17] subjected ewes to a 15% reduction in nutrient uptake for the first 70 days of gestation, and reported increased growth of the fetal side of placentomes. They hypothesized that the increase in placental tissue reflected an attempt by the fetus to compensate for a reduced nutrient supply, because increased placental villous density within the placentome allows for increased placental vasculature at the site of exchange of nutrients between the caruncle and cotyledon. Indeed, it is hypothesized that the number of placental villi formed in early pregnancy establishes placental blood volume in humans for late gestation [29]. It is important to note that uterine, placental and fetal developmental effects reported for different models of fetal growth retardation vary significantly [17, 20, 30–32]. However, regardless of the specific developmental effects, all of these phenomena, including altering the S1P signaling pathway, involve complex changes in placentome morphology that are expected to diminish fetal development through altered availability of hemotrophic support. In the present study the interplay between vascularity and nutrient availability is perhaps the underlying cause of altered amino acid composition of allantoic and amniotic fluids from FTY720 treated ewes.

It is well established that efficient placental amino acid transport is required for fetal growth [33, 34]. Allantoic fluid serves as a reservoir for electrolytes, water, sugars, proteins and other nutrients which can be absorbed by the allantoic epithelium into the fetal-placental circulation to support growth of the conceptus, whereas amniotic fluid uptake through the fetal oral cavity provides a source of polyamines that support proliferation and differentiation of intestinal epithelial cells within the fetal gut [35, 36]. Results from the current study demonstrate that FTY720 treatment reduced MET in allantoic fluid and Glu and Asp in amniotic fluid at day 60 of gestation, prior to the onset of rapid fetal growth that occurs during the third trimester of pregnancy [19]. During normal pregnancy, concentrations of Glu and Asp are much higher in the placenta than in fetal or maternal circulation [37]. Neither Glu nor Asp are delivered via the umbilical circulation in sheep, and the fetus lacks hepatic gluconeogenesis. Thus, a major source of placental glutamate is glutamine taken up by the fetal liver and metabolized to Glu used by the placenta. The decrease in Glu and Asp within amniotic fluid of FTY720 treated ewes suggests a lack of substrates for fetal metabolism.

Development of the intestine is critical for nutrient and waste exchange by the developing fetus. The decrease in Met, a precursor to polyamines, in the allantoic fluid of FTY720 treated ewes suggests a potential lack of sufficient substrate for synthesis of polyamines within the fetal gut [35, 38]. The diminished availability of polyamines could result in a decrease in enterocyte production and trafficking of molecules across the gut epithelia, as well as impair future lymphocyte trafficking and fetal growth, as evidenced by the decreased crown-rump length of fetuses from FTY720 treated ewes. Exposure to FTY720 treatment beyond what was performed in the present study (day 60 of gestation) might irreparably damage fetal-placental amino acid exchange resulting in alterations in composition and architecture of fetal organs. While long-term developmental consequences were not addressed in this study, it does appear plausible that the S1P signaling pathway serves a role in regulating fetal metabolic function and this may prove to be an important area for future investigation.

It is important to consider whether administration of FTY720 acts systemically outside the placenta. Although originally discovered as an immunosuppressant [39], FTY720 has more recently been tested for protective effects in a variety of chronic conditions, including high fat diet and obesity models, suggesting it has broader capabilities. Although FTY720 has no negative side effects in healthy individuals [40, 41], FTY720 treatments in a number of models have shown variable improvement in glucose tolerance and insulin sensitivity. Although some reports indicate FTY720 treatment lowered fasting glucose levels [42, 43], other data contradict these findings with no changes in fasting glucose [44]. Further, db/db mice fed FTY720 daily for 6 weeks displayed normalized fasting blood glucose levels [45]. In mice challenged with a high fat diet, FTY720 improves glucose tolerance, reduces plasma insulin, and increases insulin-stimulated glucose uptake, suggesting FTY720 may have therapeutic potential in treating insulin resistance [46]. Thus, a definitive effect of FTY720 on fasting glucose levels and tolerance is not clear, arguing that the effects of FTY720 on placentomal architecture and fetal development are likely not explained by alterations in glucose levels. Other possibilities that we can’t rule out at this time are that the unphosphorylated form FTY720 not only promotes reactive oxygen species generation [47], but can also enhance endocytosis of the SLC7A5 amino acid transporter, which can lead to mTORC1 inhibition [48]. Whether or not systemic or placental alterations occur in amino acid transporter expression will need to be determined in a future study.

Although we observed decreased vascularization of maternal caruncles with FTY720 treatment, we acknowledge that interpretation of the present results is confounded by the potential for FTY720 to alter functions of both the vascular and immune systems [49–52]. A focus on a vascular basis for changes in placentome architecture and fetal growth is warranted because FTY720 is a structural analog of sphingosine that interacts with and modulates the S1P1 and S1P3 receptors [14, 53], which are expressed by endothelial cells of the sheep placentome [13]. Further, there is evidence that FTY720 can act as both an agonist and antagonist of S1P signaling [14, 49, 52–61]. Our study focused on vascular changes within placentomes, it remains to be determined whether significant immunomodulatory effects were observed in the 30 day treatment period.

## Conclusions

In conclusion, we present evidence that modulation of the S1P signaling pathway results in the alteration of caruncular vasculature, placentome architecture, abundance of amino acids in allantoic and amniotic fluids, and fetal growth during pregnancy in sheep. The marked morphological changes in placentome histoarchitecture, including alteration in the vasculature, may be relevant to fetal growth and survival. It is somewhat surprising that fetal length was reduced as early as day 60, because fetal growth in sheep is greatest after day 60. The subtle changes observed in the fetuses of ewes exposed to FTY720 may indicate an adaptive response of the fetuses to cope with altered placental morphology.

## Data Availability

All data generated or analysed during this study are included in this published article (and its supplementary information files).

## Abbreviations

Ala: Alanine
Arg: Arginine
Asn: Asparagine
bAla: Beta-alanine
bFGF: Basic fibroblast growth factor
CIDR: Controlled intravaginal drug releasing
Cit: Citrulline
Cys: Cysteine
FTY720: 2-amino-2[2-(− 4-octylphenyl)ethyl]propate-1,3-diol hydrochloride
Gln: Glutamine
Gly: Glycine
His: Histidine
Ile: Isoleucine
Leu: Leucine
Lys: Lysine
Orn: Ornithine
PAS: Periodic acid-Schiff
Phe: Phenylalanine
Pro: Proline
S1P: Sphingosine-1-phosphate
Ser: Serine
SPHK1: Sphingosine kinase 1
Tau: Taurine
Thr: Threonine
Trp: Tryptophan
Tyr: Tyrosine
Val: Valine
VEGF: Vascular endothelial growth factor
